# The Dynamics of Foraging Trails in the Tropical Arboreal Ant *Cephalotes goniodontus*

**DOI:** 10.1371/journal.pone.0050472

**Published:** 2012-11-28

**Authors:** Deborah M. Gordon

**Affiliations:** Department of Biology, Stanford University, Stanford, California, United States of America; Centro de Investigación y de Estudios Avanzados, Mexico

## Abstract

The foraging behavior of the arboreal turtle ant, *Cephalotes goniodontus*, was studied in the tropical dry forest of western Mexico. The ants collected mostly plant-derived food, including nectar and fluids collected from the edges of wounds on leaves, as well as caterpillar frass and lichen. Foraging trails are on small pieces of ephemeral vegetation, and persist in exactly the same place for 4–8 days, indicating that food sources may be used until they are depleted. The species is polydomous, occupying many nests which are abandoned cavities or ends of broken branches in dead wood. Foraging trails extend from trees with nests to trees with food sources. Observations of marked individuals show that each trail is travelled by a distinct group of foragers. This makes the entire foraging circuit more resilient if a path becomes impassable, since foraging in one trail can continue while a different group of ants forms a new trail. The colony’s trails move around the forest from month to month; from one year to the next, only one colony out of five was found in the same location. There is continual searching in the vicinity of trails: ants recruited to bait within 3 bifurcations of a main foraging trail within 4 hours. When bait was offered on one trail, to which ants recruited, foraging activity increased on a different trail, with no bait, connected to the same nest. This suggests that the allocation of foragers to different trails is regulated by interactions at the nest.

## Introduction

An organism’s behavior determines its resource use and thus its ecology. The foraging ecology of ants is the result of the collective behavior that leads the colony to find and exploit new food sources. Tropical arboreal ants are diverse and abundant [1], and important in many tropical communities, often as the mutualist partners of plants [2]. The foraging behavior of colonies of arboreal ants is difficult to observe and barely studied. Stable isotope studies show that most tropical arboreal ants feed on plant and insect exudates [1], [3]. Like any ant species, arboreal ant colonies must adjust the allocation of foragers to the dynamics, in space and time, of their food sources. This collective behavior determines how far the foragers travel from nests to collect food, how the colony finds new food sources, how often colonies shift foraging areas, and how ants are allocated to different trails to get the food back to the nest. In addition, in polydomous ant species, species with multiple nests, collective behavior regulates how the ants, the brood and the resources are distributed among nests.

An ant colony’s foraging behavior influences its interactions with other species. Studies of ant community assembly suggest that differences among species in foraging behavior structure tropical ant communities [4], [5], [6]. Many studies of tropical ant communities are based on counts of ants made at baits (e.g. [7], [8], [9]). Studies of foraging behavior are essential to the interpretation of data on the species distribution of ants at bait, because species differ in how they find and respond to new food sources.

Arboreal ants face particular constraints in searching for new food sources because they must follow pathways along the vegetation [10], [11]. Because the ants are travelling along stems that bifurcate to other stems, either on the same plant or on another one, their search for food sources along branches is what in computer science is called a ‘binary tree’, involving successive choices between two alternatives [12].

Here I report on the dynamics of the foraging behavior of the turtle ant *Cephalotes goniodontus*. This polydomous species is extremely abundant in the tropical dry forest of western Mexico (Gordon, unpubl. data). *Cephalotes* is a large genus of about 115 species of arboreal ants, widespread in the neotropics [13]. The role of *Cephalotes* species in competition for nest sites has important effects on tropical community structure [14]. In many species, the major workers use the visor-shaped head-disc to guard nest entrances [13]. One remarkable species, *C. atratus*, lives high in the canopy, and workers that fall or jump out of trees can glide back on to the trunk [15]. Another, *C. macalatus*, follows the foraging trails of an *Azteca* species to exploit the *Azteca’*s food sources [16].

What *Cephalotes* ants eat has long intrigued tropical ecologists (e.g. [17] ). Davidson *et al*. [1] found that 7 species of *Cephalotes* in the Amazon were mainly herbivorous. Ants in this genus have been observed to forage for pollen (e.g. [18], [19], [20] and nectar [20], [21]. A study of *C. atratus* and *C. pusillus* in Venezuela found that these species collect nectar, homopteran secretions, and bird droppings, and that the bacteria in their digestive tract are necessary for digestion [22], [23]. It appears that the gut bacteria widespread in *Cephalotes* are involved in fixing, upgrading or recycling nitrogen [24], [25], suggesting that the ants may need only to find sources of nitrogen and of carbohydrate, but not protein.

To study the foraging behavior of the polydomous turtle ant, *Cephalotes goniodontus*, I began by determining what food sources the ants use, where they nest and where they forage. Then, to investigate how a colony of *C. goniodontus* adjusts its foraging trails, I asked: 1) How stable is a colony’s foraging area, from year to year, week to week and day to day? 2) When and on what spatial scale do colonies search for new food sources? 3) Does the allocation of foragers to a trail depend on local interactions near a food source, or is it regulated at the nest?

## Methods and Results

The foraging behavior of *C. goniodontus* was studied in the tropical dry forest of Estación de Biología Chamela, administered by UNAM, and located in the State of Jalisco, Western Mexico (19° 30′N, 105° 03′W). Studies were conducted during the wet season in July 2007, August 2009, July–August 2010, and September–October 2011. In September 2008, after a severe tropical storm (Lowell), activity of *C. goniodontus* was extremely low, and it was not possible to observe them foraging.

### 1. Natural History

#### A. Nest sites

The ants nest in cavities, some apparently abandoned cavities made by insects, in dead wood or in broken ends of dead branches, in both dead or live trees. Trees used for nests included several *Ficus* species, several *Acacia* species, *Ipomoea wolcottiana, Guapira macrocarpa,* and *Guazuma ulmifolia.* It was not always possible to observe the ants high enough in the trees to determine which trees were used as nests and which were foraging sites. Most colonies observed appeared to have many nests: foragers on linked trails, from the same colony, were seen taking food into cavities in more than one tree.

#### B. Food sources

Foragers carry many different foods back to the nest, including some rich in nitrogen such as lizard feces, bird feces, caterpillar frass, and lichen. Large numbers of ants gathered to collect bits of fungus from a leaf on the ground. Other food items carried back to the nest include small plant parts and drops of liquid carried in the mandibles. The ants forage for nectar and for plant fluids, both of which they appear to drink in place. They were seen clustered around extrafloral nectaries at the base of leaf buds, and biting on the base of the back of leaves of an *Ipomoea* vine, which may induce the growth of pearl bodies (Mark Olsen pers comm). Foragers often stop and engage in trophallaxis on the foraging trail, apparently sharing liquids obtained high in the canopy.

The ants often cluster at the edges of herbivory wounds on leaves, drinking leaf fluids. Foraging trails often led to the tops of trees showing signs of intense herbivory. For example, the largest colony observed in 2009 had a foraging trail ending in a tree of *Ipomoea wolcottiana* that showed signs of intense herbivory.

In the course of the three periods of field work in 2007–2010, the ants were offered various baits. They never recruited to protein bait, such as egg or fish, but sometimes recruited to cake, collecting crumbs to take back to the nest. Especially on dry days, they recruited to and drank from cotton soaked in sweetened hibiscus juice. The most effective bait was human urine (S. Powell, pers. comm.), which is consistent with the finding from other species in the genus that the ants’ gut bacteria make use of nitrogen [22]–[25].

### 2. Foraging Behavior and Distance Covered by a Colony’s Foraging Trails

#### Methods

A section of foraging trail was considered as a distinct trail when the ants went from a nest to a food source and back to that nest. The trail’s destination tree was the one in which the ants travelled up and down the tree and were not found in any surrounding vegetation. Two trails were considered to belong to the same colony if ants could be followed continuously from one trail to the other, sometimes interrupted by a visible nest or food source. The entire set of linked foraging trails used by a particular colony, which could link many nests and food sources, is referred to here as a ‘circuit’. Any of the foraging circuits described here could have included further trails that were not found. The colonies considered to be distinct were all separated by at least 500 m.

To determine the distance traversed by the foraging trails of a single, polydomous colony, I found for one colony in 2007 and five in 2009 the linear distance spanned by the trees used, by measuring from one tree to the next. I also found the actual length of foraging paths traversed by foraging ants by marking every piece of vegetation used within 4 m of the ground, and measuring the distance along each piece that was travelled by the ants.

To determine how long a colony uses a given set of foraging trails, the foraging behavior of six colonies was studied in July-August 2009. My assistants and I followed trails and marked with plastic flagging all vegetation wider in diameter than 5 mm, mostly twigs and stems. To mark the vegetation, we tied the flagging to a stem extending from each piece of vegetation on which the ants travelled. When the trail continued high into the canopy, we used a ladder and binoculars to follow the ants as far as we could. Often a single trail extended from the top of one tree to the top of an adjacent one.

#### Results

The ants forage in trees, and rarely descend to the ground. When moving along a trail, between food sources and nests, the ants follow the trails exactly and do not deviate. When exploring, apparently searching for new food sources, ants go up and down every possible stem and branch in the vegetation, and travel all over leaves.

Foraging trails move along a convoluted path of vines, twigs, and branches. On a hot day (3 Aug 2009) we measured the time it took 20 ants to travel along 38 cm of trail, 10 ants toward and 10 away from a nest, and found an average speed of 4.39 cm/sec (SD 0.62).

In all cases, distinct foraging trails of the same colony met at a nest. It appears that most trails included more than one food source, because groups of ants were always observed at food sources, while ants coming into a given nest carried many different kinds of food. This indicates that groups of ants from different food sources all returned to the same nest.

Because the foraging trails follow the diverse shapes of different kinds of vegetation and involve so many transitions from one piece of vegetation to another, the distance travelled by ants is much longer than the linear distance traversed, by a factor of 2 to 5. For example, in one colony, a foraging trail that went from one tree with a nest to another tree at a linear distance of 8 m had a length of 39.6 m, with 38 transitions from one separate piece of vegetation or different branch of the same woody plant to another. The average distance between transitions from one piece of vegetation to another was 10 cm. In another colony, a foraging trail involving 3 trees that spanned a linear distance of 12 m had a length of 49.5 m, with 29 transitions from one separate piece of vegetation or different branch of the same woody plant to another. In a third colony, the ants travelled on 28 m of path to traverse a linear distance of 15 m.

Foraging trails follow the smallest pieces of vegetation. For example, on a vine with curling tendrils, the ants followed the spiral of the tendril to get from the vine to a branch of another plant. The trails make use of extremely ephemeral connections between pieces of vegetation. For example, one trail used an edge of a leaf in contact with a branch to get from one small branch to another. This junction was used for two days, and on the third there was a new pathway around it. Another colony’s trail used a broken branch, tangled in vines, that was leaning against a tree. When the wind blew the branch away, the connection was lost. Ants that arrived at the tree when the branch had been blown out of place waited at the gap, like passengers waiting for a ferry, until the wind died down and the branch came back, and then stepped onto it.

For the six colonies found in 2009, complete trails were observed in five; in the sixth there were ants exploring leaves but no nest was found. The numbers of large trees used ranged from 3 to 8. The smallest circuit for a colony had a single trail from one nest to a tree with a food source, using 3 trees spanning 2.5 m of linear distance, with 10 m of foraging trail, while the largest had a circuit with 3 distinct trails linked to 2 different nests and at least 3 food sources, using 7 large trees spanning 19 m in linear distance and about 100 m of foraging trail.

It appears that the ants are marking the trail with a chemical cue. When an ant comes to a junction and there are no other ants nearby, it explores the junction with its antennae and then goes in the direction used by the last ants to traverse that junction. Ants may also use information based on the frequency of antennal contact; when ants travelling in opposite directions meet at a junction, such as a fork in a branch, they antennate each other.

### 3. Stability of Foraging Circuits

#### Methods

To evaluate the day-to-day stability of foraging trails, the six colonies studied in 2009 were checked daily for 4–8 consecutive days, noting all changes in foraging trails from the previous day.

To evaluate the year-to-year stability of foraging circuits, in 2010 I searched the areas used by the six colonies studied in 2009, as well as further vegetation about 30 m, about twice the distance of the longest trail observed, on all sides surrounding the marked area.

#### Results

From day to day, the colony often uses the same trails. Of the six colonies for which foraging trails were observed in 2009 from day to day, two used exactly the same path, travelling on the same pieces of vegetation, for 8 days. The only change occurred when, in one colony’s circuit, a branch that was part of the trail broke off, and the trail changed to get around this gap. In another two colonies, part of the same trail was used on all days, but both colonies also developed new trails in the course of the 8 days. In one of the remaining two colonies, the ants were exploring leaves in the same location for 2 days, and after that, no ants were seen there. Perhaps there were trails elsewhere and the ants were merely scouting in the area where they were observed. The sixth colony was found in a nest in a broken branch on the ground. The ants moved from the dead branch on the ground into a nearby tree, apparently part of their original trail, and for the next 4 days used the same trail, which originated from this tree.

On the scale of weeks and longer, trails are abandoned and new ones are formed. One cause is damage to the nest or to vegetation supporting the trail. In this situation a new trail forms nearby. On four occasions we found nests in recently fallen branches on the ground the morning after a storm. At first the ants went back and forth on the branch, eventually onto the ground, and then, once the ants located an existing trail in a tree, they abandoned the nest on the ground and moved back onto the trail.

Interactions with other species influence the stability of foraging trails. The presence of ants of other species, especially species of *Azteca*, *Crematogaster*, and *Pseudomyrmex*, was sufficient to deter ants from using a trail. In one observation, the arrival of many workers of an *Azteca* species at a sugar-water bait to which *C. goniodontus* had recruited caused the *C. goniodontus* ants to retreat to their nest. In another, one worker of *Pseudomyrmex* sp. walked back and forth for more than an hour on a branch that was part of the *C. goniodontus* trail up a tree with a nest. The *C. goniodontus* used an alternative trail, avoiding that branch, but went back to that trail the next day when no *Pseudomyrmex* were present. No naturally-occurring interactions with other conspecific colonies were observed.

Abandoning routes, and starting new ones, leads the location of the foraging circuits to shift gradually over time. Of six colonies observed in July-August 2009, only one persisted in approximately the same place in 2010. This may have been a different colony in the same location. In this case, the ants were not in the same trees, but in other trees within about 20 m. A nest had been found in 2007 about 20 m from the 2009 location, but not seen at that location in 2008, when the activity of *C. goniodontus* was low.

### 4. Searching for New Food Sources

#### Methods

I examined how frequently, and how far from existing trails, ants search for new food sources. Baits were placed on a branch that was at least 3 bifurcations, such as a stem or new piece of vegetation, away from an existing trail. On Oct 5 2011, 5 baits were placed, each 3 junctions from the trail, at each of 3 colonies. The distance from the main trail to the bait was less than 0.5 m in linear distance. The bait was a ball of cotton soaked in human urine, attached to the vegetation with wire. Baits were placed between 1100 and 1300, checked again 3 and 5 hours later, and then removed. The sites where baits had been attached were checked again the following day. At each check, a count was made of the number of *C. goniodontus* ants on the cotton ball bait, and a snapshot count was made of the number of *C. goniodontus* ants on the route spanning 3 junctions from the trail to the bait.

#### Results

Ants quickly found new food sources that were 3 bifurcations away from the main trail. Within 4 hours, ants were on both the bait and travelling from the main foraging trail to the bait for 5 of 5 baits in 1 colony, 3 of 5 baits in 1 colony, and in 2 of 5 baits in 1 colony. In the latter colony there were many *Pseudomyrmex ants* at the 3 baits with no *C. goniodontus*. Baits were removed after 4 hours. The following day there were no ants at the sites of any of the 5 baits in any of the 3 colonies, although the main foraging trails were still in use.

### 4. Allocation of Foragers to Trails

#### a. Methods: Undisturbed colonies

**Figure 1:**
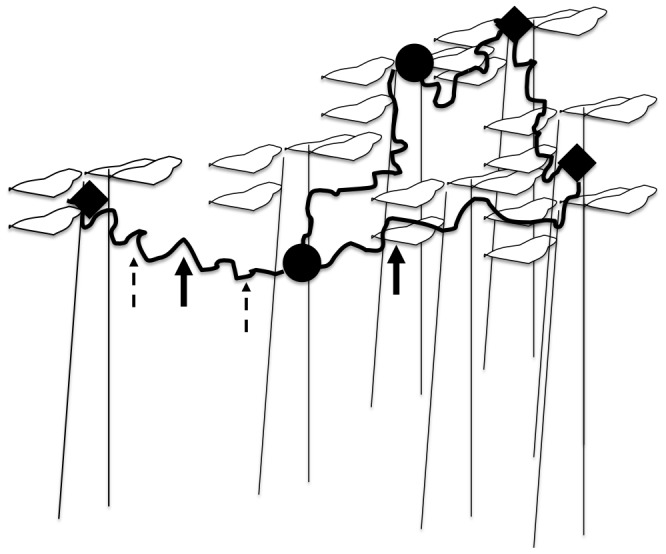
Illustration of a foraging circuit of a colony. The ants forage in dense vegetation that fills the spaces between trees; only a few trees are sketched here. Curving lines show foraging trailsthat connect a nest (filled circles) and one or more food sites (filled diamonds). Solid arrows illustrate two sites at which ants were marked, released and observed on differenttrails, one on the trailon the left, and the other on a second trailon the right. Dashed arrows illustrate two sites in a different colony at which ants were marked, released, and observed on the sametrail. The vertical lines represent tree trunks. This figure illustrates one possible configuration of many different ones observed.

To determine how individual ants are allocated to foraging trails, and whether each ant travels the entire foraging circuit, observations were made of marked ants in July 2010. In each of three colonies, we collected 50–150 ants at each of two sites, marked them with a unique color corresponding to the site at which they were collected, and released them at that site. Ants were marked with acrylic paint on the head, thorax and abdomen, and released within 2 h. We saw no effect of marking on ant behavior and, once the paint was dry, no unusual response to marked ants from unmarked nestmates. Of the three colonies in which ants were marked, one colony (colony 10) apparently travelled only in a single trail from a single nest to a food source and back. The other two colonies (8 and 14) were larger, and each had two distinct trails that met at a nest. In colony 10, with a single trail, two groups of ants were collected, marked and released at two sites 2 m apart along the same trail (represented by the two dashed arrows on the left side of Fig. 1). In the other two colonies, 8 and 14, with two trails, ants on each trail were collected, marked with a unique color for each trail, and released at the same site. The two sites are represented by two solid arrows in Fig. 1, one on the trail shown with a dotted line on the left, and one shown with a solid line on the right.

In all colonies, the sites where ants were marked and then observed were within 5 m of a nest. In colony 10, the two collection sites were along a single trail that linked four large trees, one with a nest, that spanned a linear distance of 6 m; collection site 1 was 1 m from the nest tree and collection site 2 was 4 m from the nest tree. In Colony 8, there were two trails that used five large trees spanning a linear distance of 10 m. The collection site 1 was 4.5 m from the nest, in the middle of a trail that extended to a tree a further 2 m from the observation site, while collection site 2 was at the end of another trail 4 m from the nest. In Colony 14, there were two trails that involved six large trees spanning a linear distance of 6 m and met at a nest. The collection site 1 was on a tree 1 m from the nest tree, while collection site 2 was on a tree 1 m away on the opposite side of the nest tree, in the middle of a second trail that extended into a tree 5 m from the nest tree.

Observations were made at each site to determine if ants of a given color were most likely to be seen at the site at which they were marked. We made a set of 10–11 consecutive 5 min observations on each of 2 days for Colony 10, 3 sets on each of 3 days for Colony 8, and 3 sets of observations, one in the morning and one in the afternoon of one day, and one on the next day, for Colony 14. In all colonies, observations were made simultaneously at sites 1 and 2. The observer counted the numbers of ants of each color, and the numbers of unmarked ants, of *C. goniodontus*, passing an imaginary line on a branch or trunk, during 5 min. All of the observations were on trails long enough that it would take an ant much more than 5 min, and possibly more than the 55 min duration of the set of observations, to return to the same place.

To determine whether individual ants tend to stay in the trail in which they were foraging when marked, I found for each day the total numbers of marked ants of each color observed at each site, over all 5 min observations on that day. For each day I tested whether the distribution of marked ants of color 1 and color 2 differed at sites 1 and 2, using Fisher’s exact tests for all sets of observations except one, for which numbers of ants were large enough to require the use of a chi-squared test.

#### Results: Undisturbed colonies

**Figure 2:**
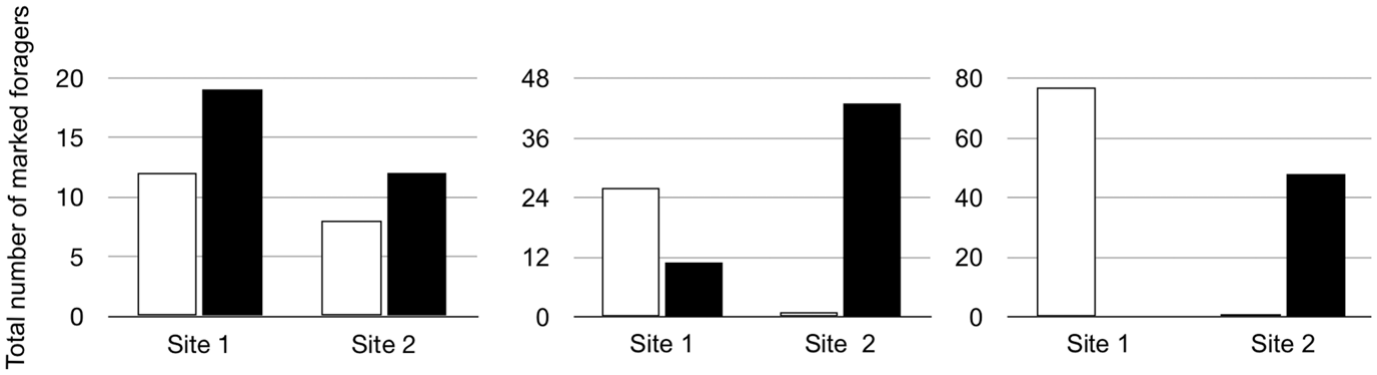
Distribution of marked ants on foraging trailss. The y axis represents total numbers of marked ants observed passing a point on a trail on a given day in 11 5-min counts. Open bars, ants marked at site 1; Filled bars, ants marked at site 2. Each graph shows representative results from a differentcolony, each on one day of observation. Left, Colony 10 with a singletrail; Middle, Colony 8 with twotrails; Right, Colony 14 with twotrails.

Ants within a single trail travel the entire trail. Ants marked at one site along a single trail were later seen at another site on the same trail. In colony 10, in which two groups of ants were collected at each of two sites, and each marked a unique color, along the same trail, there was no significant difference in the distribution of the numbers of ants of the two colors at the two sites (Day 1 20 Jul 2010, Fisher’s exact test, p = 0.6, Day 2 22 Jul 2010, Fisher’s exact test p = 1; Fig. 2).

When a foraging circuit consisted of more than one trail, individual ants tend to travel on only one trail, and not to travel the entire foraging circuit. Marked ants tended to travel only in the trail in which they were originally collected. Ants were significantly more likely to appear on the trail at the site where they were marked than on the other trail (Fig. 2). There were significant differences in the distribution of marked ants of the two colors on the two trails: Colony 14 Day 1A 22 Jul 2010 10:15, p<0.0001; Day 1B 22 Jul 2010, 17:30, p<0.04; Day 2 23 Jul 2010 p<0.0001, all Fisher’s exact test; Colony 8 Day 1 18 Jul 2010, p<0.03, Fisher’s exact test, Day 2 19 Jul 2010 p<0.00001, chi-square test (n too large for Fisher’s exact test), Day 3 22 Jul 2010 p<0.00001, Fisher’s exact test).

#### b. Methods: Allocation of foragers in response to new food sources

To test whether new food sources alter the allocation of foragers to particular trails, ants were marked and observed on trails with bait. In each of 3 colonies, observations were made at 2 sites along 2 distinct trails that met at the same nest. Ants on each trail were marked as above, with a unique color for each trail, one called the bait trail and the other “Trail X”. The baits were cotton balls soaked in urine and attached to a branch with a wire, as above. Numbers marked were: Oct 1 2011, Colony 20, 60 on bait trail and 30 on Trail X;Oct 3 2011, Colony 21, 75 on each trail, 10-3, Colony 22, 50 on each trail. Observations were made the day after marking for all colonies: Oct 2 2011 for Colony 20 and October 4 2011 for Colony 21 and Colony 22. We counted the number of ants passing a point on the trail in 5 min: 1) at bait, 2) on the same trail as the bait, closer to the nest than the bait 3) on the other trail that had no bait. We made 10 5-min counts, in the hour before bait was placed, immediately after the bait was placed, and then beginning 3 hrs later with the bait still present. Observations of the 3 colonies began at 1000 and continued until 1700. To compare the change with time of day in numbers on trails when no bait was available, we observed 3 colonies, of which 2 were the same ones used in the bait experiment, from 12-1 and 1600–1700 on Oct 14 2011.

To determine whether foraging activity changed on the trail without bait before and after the bait was placed, I found the ratio of the foraging rate summed over all 10 counts 3 hours after to before bait was placed, and determined whether the mean ratio differed from zero. and used Fisher’s exact test to compare number of ants on other trail. To compare the change with time of day in numbers on trails when no bait was available, I used Fisher’s exact test to compare the number of ants on each trail from 1200–1300 and 1600–1700.

To determine whether individual foragers changed trails in response to the bait, I used Fisher’s exact test to compare the number of marked ants on each trail before and 3 hours after the bait was provided.

#### b. Results: Allocation of foragers in response to new food sources

**Figure 3:**
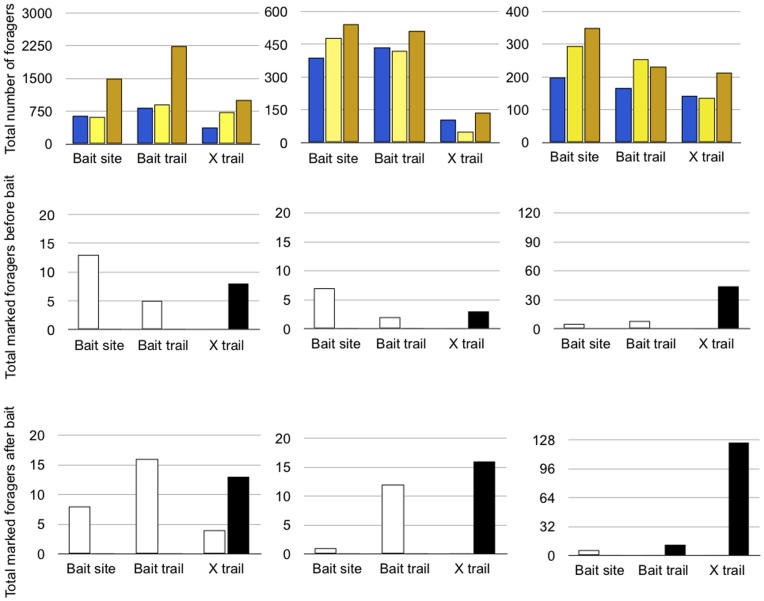
Increase in foraging rate and distribution of marked ants in response to bait. Each column shows the results from one colony on one day. The top row shows the total number of foragers observed passing a point on the trail in 10 5-min counts, for a total of 50 min, at the site of the bait, on the trail with bait at a site between the nest and the bait, and on another trail without bait designated as “X” trail. Blue bars show numbers observed before bait was placed, yellow bars show numbers observed in the 50 min after bait was placed, and gold bars show numbers observed 3 h after bait was placed. The middle and bottom bars show the results for marked foragers in the same observations. Open bars show ants marked on the bait trail, solid bars show ants marked on the other A trail. The middle row shows the total number of marked foragers on the two trails of the indicated colony in the 50 min before bait was placed. The bottom row shows the total number of marked foragers on the two trails summed for the two observations immediately after and 3 h after bait was placed.

When there was bait on one trail, to which ants recruited, foraging activity increased on a different trail, with no bait, connected to the same nest (Fig. 3). The foraging rate on the trail without bait (designated in Fig. 3 as “Trail X”) was higher 3 hours after bait was placed on the other trail in all 3 colonies. The ratios of numbers of foragers observed passing a point on the alternate trail after bait was placed to numbers observed on that trail before bait were 2.68, 1.31 and 1.5; the mean (SD) was 1.83 (0.74) so more than 2 standard deviations from 0. In the absence of bait, numbers on the trails did not increase during the same time of day, from 1200–1300 to 1600–1700; the same ratios for 6 trails, 2 per colony, ranged from 0.6 to 1.1; the mean (SD) was 0.89 (0.19) so not significantly different from zero.

Ants tended to remain on the trail on which they were marked. Although the number of ants increased on both trails when bait was placed on one of the trails, marked ants did not switch from one trail to the other, in any of the 3 colonies (Fisher’s exact test for all 3 colonies, p<0.0001; Fig. 3). Total numbers of marked ants observed vary in the three colonies because they differed in the number of ants that were marked.

## Discussion

The collective foraging behavior of a turtle ant colony allows it to collect ephemeral, patchy resources, including plant fluids such as nectar and sap from herbivory wounds, and bird and lizard feces. Ants travel in a circuit that consists of a series of trails from nest source to food source, with more than one trail from a given nest, and other sections of trail that lead from one nest to another. The resources used by *C. goniodontus* require foraging behavior that allows them to maintain a steady flow of traffic at the food source, and to match the numbers of ants to the rate of flow of plant nectar or fluid in the phloem [26]. The foraging circuit is extremely stable in the short term, from day to day. The allocation of individuals within the foraging circuit allows the colony to persist at food sources. Individual ants tend to stay on the same trail and not to complete the entire circuit, regardless of the presence of bait (Figs. 2 and 3).

It appears that certain ants are allocated to collect a certain resource on a trail that can persist for many days. This trail fidelity makes the foraging circuit more resilient to changes in the fragile links in vegetation along which the ants travel, and to damage to their nests in dead wood. Damage in one trail does not necessarily impede foraging in another, because the ants on one trail can continue foraging while the ants on a damaged trail find a new trail. Such resilience in the face of disturbance may account for foraging circuits in other ant species. For example, the polydomous Argentine ant (*Linepithema humile*) uses many nests linked by trails that pass by food sources [27].

Individual fidelity to a foraging trail or foraging site occurs in many ant species (e.g. [28], [29]), and is probably associated with long-lasting resources. Like the red wood ant that forages on very permanent trails to tend stable populations of aphids [30], *C. goniodontus* foragers on one trail are unlikely to switch to another. By contrast, in harvester ants, which forage for seeds that are scattered by the wind so that patches are ephemeral [31], foragers easily switch trails when a food source appears in a new location [32].

Local interactions at nests, which function as the nodes that connect distinct trails, apparently regulate the intensity of foraging behavior. When a new food source appears on a foraging trail, more ants forage on other trails connected to the same nest (Fig. 3). However, ants marked on one trail did not use the other (Fig. 3). This means that foraging activity increased on the trail without bait because more ants were recruited from the nest. Further work is needed to discover how this is done. It is not clear whether recruitment includes any spatial information about the location of food sources. Foragers returning to a nest are often groomed extensively by ants waiting near the nest entrance, and this may provide some cue to the odor of the food sources visited by the returning foragers. The stimulation of activity on one trail due to a new food source on another trail suggests that resources, such as nectar, tend to be available simultaneously in different places in adjacent vegetation, and thus the discovery of a new resource on one trail is often associated with a similar discovery on another trail.

The persistence of trails, and the formation of new ones, allow colonies of *C. goniodontus* to collect resources that are patchy and persist for several days. Most of the resources collected were plant derived. Nectaries on buds or at the base of leaves, nectar in flowers, and phloem extracted on leaf wounds, may all be available for days at a time. From one day to the next, the colony uses the same path to visit the same resource, apparently feeding on the same sources until they are depleted or until the ants are forced to move because of interference by other species. Although forager fidelity to a given trail is high, there is also continual searching at least 3 junctions off the trail that allows the colony to find new resources within several hours. The modification of foraging trails leads to a continual shift, on the timescale of months, in the colony’s foraging circuit. From year to year, I found only 1 of 5 colonies within 30 m of their location the previous year. Further work is needed to investigate the foraging activity of this species in the dry season when most trees have lost their leaves.

New trails to baits were abandoned after the bait was removed. Further work is needed to determine how the decay of volatile trail pheromone and other interactions at the nest combine to stop the ants travelling to a food source when it is depleted.

Colonies probably modify existing trails to reach new nest sites as well as new food sources. Nest site limitation is an important ecological pressure for many species of *Cephalotes* [13], [14], [33]. Nest sites for *C. goniodontus* are ephemeral, since they nest in dead branches that often break and fall to the ground.

Colony sizes are certainly in the hundreds of workers and may extend to thousands in the largest colonies. The ratio of marked to unmarked ants provides a rough estimate of colony size. In one observation of ants at colony 8 in 2010, 23 percent of the ants travelling past one site were marked ants of a given color, and there were no marked ants of the other color. Since 100 ants of that color were marked, this suggests that about 400 ants were travelling on that trail. On the other, longer trail, only 3 percent of the ants observed to travel on that trail were marked. Since 150 ants of that color were marked, the same reasoning would suggest that there were several thousand ants on the trail. In the largest colony observed in 2009, there were 10–30 ants travelling along each meter of trail, and the entire circuit measured extended at least 100 m in path length, leading to an estimate of 1000–3000 ants foraging with a larger overall colony size because some ants must remain inside of nests.

The consumption of plant fluids by ants may have an important impact on tropical dry forest communities [6], [26], [34], [35]. For example, *C. goniodontus* collects plant sap from the edges of herbivory wounds. This suggests that the ants thus increase the cost to plants of herbivory, so that herbivory may indirectly promote ant populations. The use of nectar by ants that do not defend the plant may influence evolutionary pressure on mutualistic interactions [20]. *Cephalotes goniodontus* also collects caterpillar frass and lizard feces. This suggests that if their gut bacteria are similar to those of other *Cephalotes* species [22], [24], the bacteria may be involved in recycling nitrogen from the urea and uric acid in animal waste, as well as upgrading the amino acids in plant sap.

This work is a first step in investigating how the foraging behavior of *C. goniodontus* determines its ecological role in the tropical dry forest. The collective foraging behavior used by *C. goniodontus* allows them to search for patchy and ephemeral resources through bifurcating pathways. Its resource use depends on how, over time, the foraging circuit changes in response to damage to the vegetation supporting the trail, the depletion of food sources, the discovery of new sources, and interference from other species.
